# Home-based geriatric rehabilitation after inpatient rehabilitation: a redesign and feasibility study

**DOI:** 10.1186/s12877-025-06043-z

**Published:** 2025-06-02

**Authors:** Astrid D. Preitschopf, Margriet Pol, Bianca Buurman, Marije Holstege

**Affiliations:** 1 Department of Medicine for Older People, Amsterdam UMC, location Vrije Universiteit Amsterdam, de Boelelaan 1117, Amsterdam, The Netherlands; 2 Amsterdam Public Health, Aging & Later Life, Amsterdam, The Netherlands; 3 Department of Research GRZPLUS, GRZPLUS; Omring and Zorgcirkel, Hoorn, The Netherlands; 4 Department of Research Omring, Hoorn, The Netherlands; 5 Research group occupational therapy: Technology and Participation, Faculty of Health, Amsterdam University of Applied Sciences, Centre of Expertise Urban Vitality, Amsterdam, The Netherlands; 6 Research group geriatric rehabilitation, Centre of Expertise Prevention in Health and Social Care, Faculty of Health, Sports and Social Work, Inholland University of Applied Sciences, Amsterdam, Netherlands

**Keywords:** Blended eHealth, Co-creation, Community care nursing, Feasibility study, Goal setting, Healthcare professionals, Home-based geriatric rehabilitation, MRC framework, Older adults, Support system

## Abstract

**Background:**

There is a shift from inpatient to home-based geriatric rehabilitation (HBGR), and potential benefits are demonstrated. Previously, a theoretical HBGR model, version 1.0, has been developed, outlining its essential components. However, clear guidance on the practical design and organisation of HBGR in everyday practice is still lacking. Therefore, determining the optimal design for this complex intervention is essential for its successful implementation in daily practice. The objective of this study is to redesign the theoretical HBGR trajectory and assess its feasibility, acceptability, and usability from both patient and professional perspectives.

**Methods:**

A redesign and feasibility study based on the MRC framework was conducted in a Dutch skilled nursing facility using the MRC framework in co-creation with eleven healthcare professionals and four patient representatives. The HBGR trajectory 1.0, comprises four building blocks (structure, process, environment, and outcomes) based on the Post-Acute-Care rehabilitation quality framework. Version 1.0 was redesigned during the development phase and subsequently pilot-tested in daily practice during the feasibility phase. Adjustments were made based on semi-structured interviews with ten patients and (interim) evaluations.

**Results:**

The HBGR trajectory 1.0 has been redesigned into version 2.0. It contains eleven elements: individualised goal setting, providing HBGR is the default unless otherwise indicated, an information letter, blended eHealth, mapping the patient’s living environment, stimulation support from informal caregivers, collaboration with community care nursing, rehabilitation coordination, central planning, therapy at home, and online multidisciplinary evaluation. Version 2.0 was enthusiastically endorsed by patients, patient representatives, and professionals, who found it feasible, acceptable, and usable in daily practice.

**Conclusion:**

The HBGR trajectory 1.0 was adapted, tested, and finally redesigned into version 2.0. The study revealed that involving patients, their representatives, and healthcare professionals was critical to garnering support and facilitating implementation. Key developments align with global trends and include the successful integration of eHealth with traditional treatment methods, enhanced collaboration and knowledge sharing among community care nurses, and increased involvement of informal caregivers in rehabilitation. This redesigned HBGR trajectory is ready for evaluation and implementation in follow-up effectiveness research.

**Supplementary Information:**

The online version contains supplementary material available at 10.1186/s12877-025-06043-z.

## Background

Worldwide, geriatric rehabilitation (GR) faces the challenge of ensuring long-term sustainability, given the increasing number of older people and expected labour market shortages [1]. GR must prioritise efficiency to guarantee that high-quality care is available for all older adults.

In response to these challenges, there is an increasing focus on providing GR in outpatient settings, at home, or in the patient’s residence using a specialised team, following inpatient GR, referred to as home-based geriatric rehabilitation (HBGR) [2–5]. The potential benefits of HBGR for older adults have been demonstrated, including improved performance in activities of daily living, regaining independence, a decreased risk of falling, and an improved quality of life [6–9]. Moreover, the professional field generally agrees that GR is preferably performed in an ambulatory or outpatient setting, as demonstrated by the international Delphi study of van Balen et al. [3].

Previous research by Preitschopf et al. [10, 11] provided the context and building blocks for an evidence-based theoretical HBGR trajectory, referred to as version 1.0. This trajectory outlines the patient’s care path from the inpatient GR phase to continued GR care at home. It identifies the structure, process, and environmental components that may influence the HBGR outcomes and are essential for redesigning HBGR 1.0 into an optimal trajectory suitable for daily practice. The core building blocks elements of HBGR version 1.0 include, e.g. an inpatient start of HBGR, the integration of patient-oriented eHealth solutions with traditional face-to-face treatments (blended care), structural collaboration and integration with community care (CC) nursing, education for both patient*s* and informal caregivers, and a designated case manager as a central contact person [11]. Previous studies have examined a comprehensive rehabilitation approach aimed at enhancing the independent functioning of elderly individuals following hip fracture [12, 13]. However, no clinical studies have specifically addressed the implementation and development of the various HBGR 1.0 elements within the entire GR target group, without differentiating between specific diagnoses. Additionally, despite the need for a structured and evidence-based trajectory, there is currently no clear guidance on how HBGR should be designed and organised in everyday practice [10, 14, 15]. According to Prins et al. [16], this lack of clarity may hinder the successful implementation of HBGR. It could also contribute to international diversity in the execution of HBGR, as demonstrated by Grund et al. [17]. More clarity and consensus on the organisation of home-based GR could promote global collaboration and enhance research on home-based GR.

Designing and implementing an HBGR trajectory is not straightforward. It is a complex intervention [18] with many different organisational elements and stakeholders, such as older adults with complex and multiple pathologies and professionals from various disciplines across settings. This requires a lot of flexibility, expertise, and skills from everyone involved to complete the rehabilitation treatment. The Medical Research Council (MRC) framework can be used to redesign [18] a complex intervention using distinct phases that can be followed in an iterative process and from different perspectives. By doing so, it is possible to stimulate a better connection to daily practice because of the (early) involvement of the various stakeholders, which can improve the final implementation of an intervention.

This study aims to redesign a home-based geriatric rehabilitation trajectory for older adults living at home after inpatient rehabilitation into a feasible, acceptable, and usable trajectory by redesigning and testing its feasibility in a real-world setting and in co-creation with three stakeholder groups (patients, patient representatives, and healthcare professionals).

## Methods

### MRC framework

**Fig. 1:**
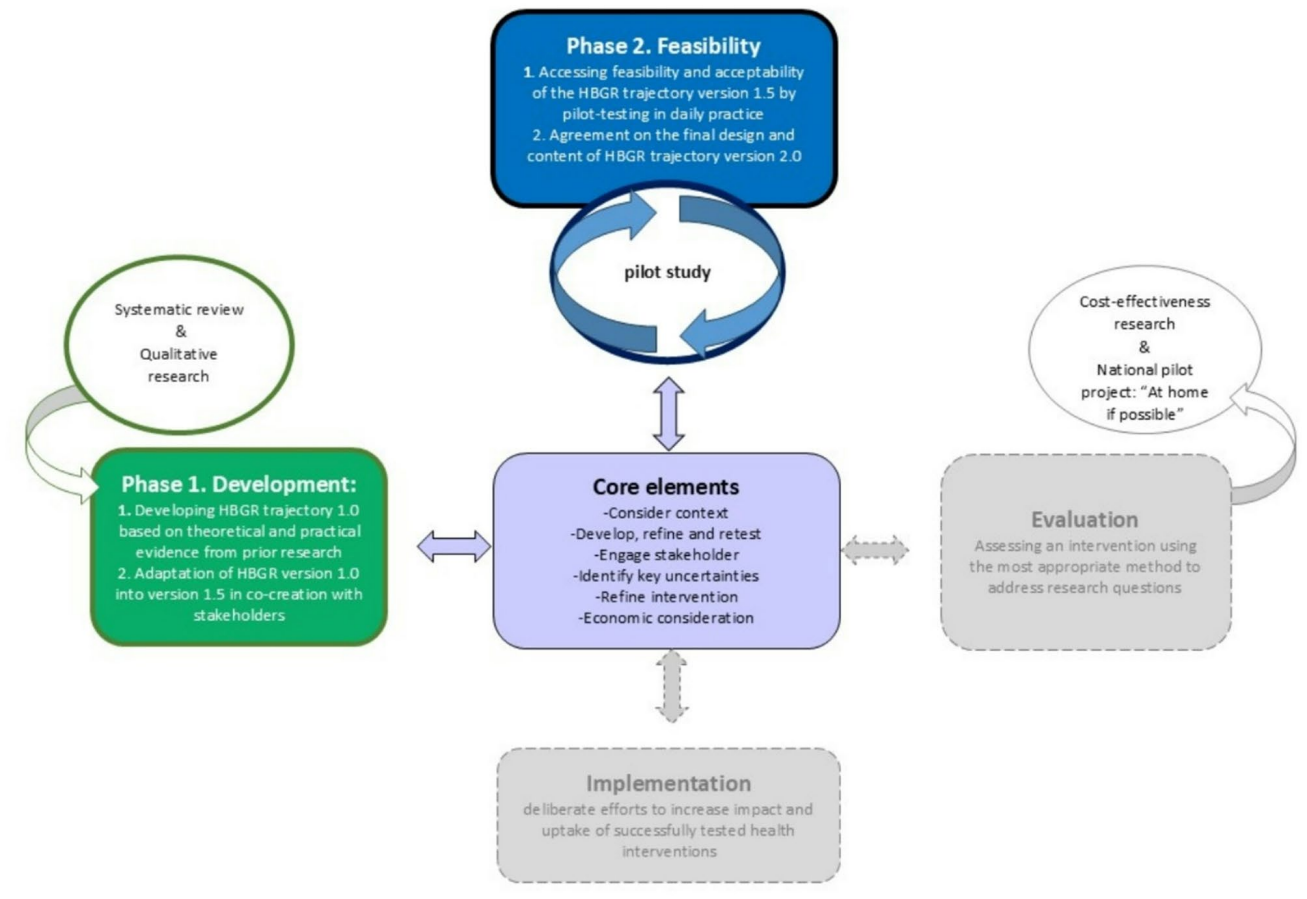
Context of the study within the MRC framework for the evaluation of complex interventions (Skivington 2021 [18])

This study used the MRC framework for developing and evaluating complex interventions as guidance (Fig. 1), as updated by Skivington et al. [18]. The framework comprises four phases that can be followed in an iterative, non-sequential process. At each phase, you refer back and forth to the central core elements, determining whether you can proceed to the next phase or back to the previous one. Our study will focus on the development and feasibility phases. The development phase served as the preparation phase, during which the HBGR trajectory was redesigned. In the following feasibility phase, the HBGR trajectory version 1.5 was pilot-tested in daily practice by gradually applying the organisational elements (adapted in the development phase), performing (interim) evaluations and adjustments, with redesign version 2.0 as the outcome. The evaluation phase (focused on assessing a redesigned HBGR trajectory through cost-effectiveness research and a national pilot project) and the implementation phase (focused on the practical application and consolidation of a well-tested HBGR design) are outside the scope of this study and will not be discussed here [18].

### HBGR version 1.0

The HBGR trajectory 1.0 was developed based on theoretical and practical evidence from our prior research [7, 8] (see Appendix 1). As this topic has been discussed in detail elsewhere, we provide a brief summary.

HBGR version 1.0 consists of four building blocks: structure, process, environment, and outcome, each comprising specific elements:

(1) Structure, which focuses on the organisation of HBGR and incorporates closer links with integrated care. (2) Process, which involves a patient-centred, multidisciplinary network, a well-coordinated multidisciplinary team dedicated to the patient’s care at home, and supported by patient-oriented, blended eHealth applications. (3) Outcome of HBGR, which is focused on regaining patients’ quality of life and autonomy. (4) Environment, which involves the stimulating aspects of the physical and social rehabilitation environment. HBGR 1.0 serves as the initial foundation and input for this feasibility study.

### Study design and setting

A redesigning and feasibility study was conducted with an action-oriented, iterative approach [19–21], allowing continuous improvement and adaptation. To gather real-life experiences and ensure a collaborative and patient-centred approach, we employed co-creation principles, which align with the Experience-Based Co-Design (EBCD) methods [19–21], thereby improving healthcare processes by integrating patient and professional experiences. This approach ensures that all stakeholders actively participate and share in the decision-making process.

We followed two phases according to the MRC framework [18] to redesign the existing HBGR trajectory 1.0 by adapting it and testing its feasibility. The study involved various qualitative methods, which were applied alternately: (1) two co-creation sessions [20] with patient representatives (*n* = 4), and healthcare professionals (*n* = 11). By applying co-creation principles, we ensure that all stakeholders are actively involved and share in the decision-making process. (2) five working group meetings [20] with seven professionals who were multidisciplinary treatment team members and actively participated during the pilot phase. (3) pilot-testing the intervention in a real-world setting [18, 20], and (4) semi-structured patient interviews (*n* = 10) [22]. The content and topics were based on the interview guide used in the previous qualitative study [11] (see Appendix 1).

This research was conducted from October 2023 to February 2024 in a geriatric rehabilitation department with 22 beds at a skilled nursing facility (SNF) in the Netherlands. The study is part of the national living lab “Better@home: development, implementation, and evaluation of home-based geriatric rehabilitation after inpatient rehabilitation” [11]. This living lab is a partnership between three regions in the Netherlands, in which two to three SNFs participate in each region.

### Participants and data collection

#### Participants

The study included three different groups of stakeholders: (1) Older adults (*n* = 10) who were admitted to a geriatric rehabilitation department in an SNF, were eligible for and underwent an HBGR trajectory (further called patients). After providing informed consent for participating in this study, patients were interviewed by AP within one week of completing the HBGR trajectory; (2) Patient representatives (*n* = 4) who were either former patients or informal caregivers, and had experience with HBGR or had gone through the home-based rehabilitation process. They were invited to think about HBGR developments critically and participated in the co-creation meetings; (3) Healthcare professionals (*n* = 11) from the participating SNF, including a physiotherapist, occupational therapist, speech therapist, dietician, elderly care physician, nurse specialist, GR nurse, CC nurse, psychologist, social worker, GR department manager, and head of the scientific research department [3]. The eleven professionals participated in two co-creation sessions, of which seven actively participated in the working group. Tasks were divided among the working group members, with a coordinator ensuring everyone followed their agreements.

Two phases were conducted to collect the data (Fig. 1) following the MRC framework [18]. Firstly, in the development phase, the existing HBGR trajectory version 1.0 was redesigned into version 1.5. Secondly, the feasibility phase, in which version 1.5 was pilot-tested in daily practice, leading to the development of version 2.0.

**Fig. 2:**
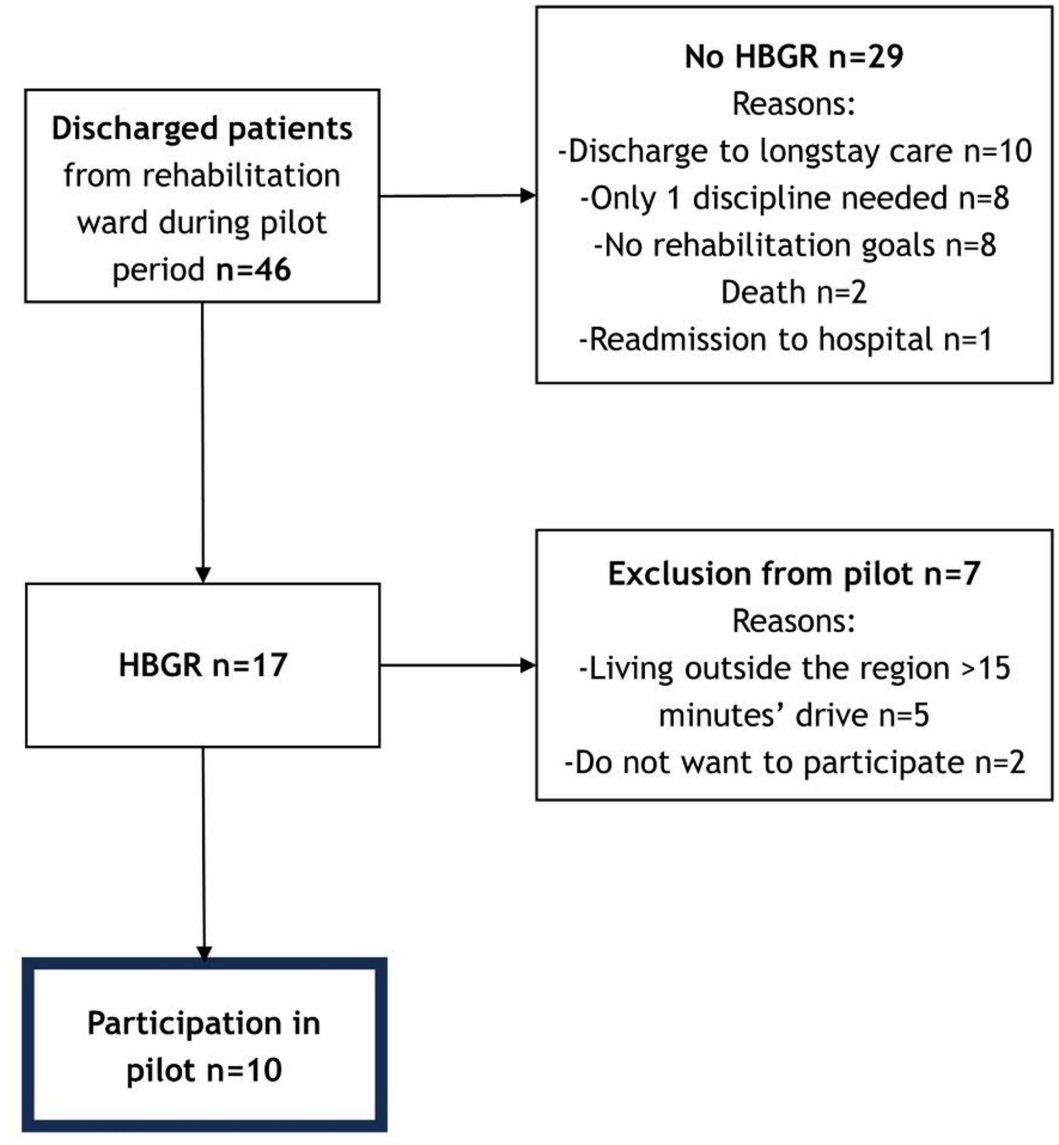
Flowchart patient participation; inclusion and exclusion reasons

#### Development phase

The initial HBGR trajectory version 1.0 underwent further adaptation during the development phase, with active involvement from healthcare professionals (*n* = 11) and patient representatives (*n* = 1) through an interactive co-creation group meeting [20]. A description of the different meetings is shown in Appendix 2. The co-creation meeting aimed to introduce the HBGR trajectory version 1.0, gather input from various stakeholders, identify areas for further development, and reach agreements on the pilot process in terms of content and task division. This phase involved successive additions to the HBGR trajectory 1.0 until the refined HBGR version 1.5 was ready for pilot testing in phase 2, the feasibility phase.

#### Feasibility phase

In this second phase, HBGR trajectory version 1.5 was pilot-tested [18, 20] in daily practice with ten patients who were eligible for and underwent an HBGR trajectory and gave informed consent to participate in this study. The experiences of the ten included patients were gathered through a 45-minute interview conducted by AP regarding home rehabilitation [22]. The semi-structured interviews (see Appendix 1) occurred at the patient’s home within three weeks after finishing the HBGR trajectory. The experiences were included in the interim evaluations and adjustment of the HBGR trajectory. The working group gradually implemented the HBGR elements to evaluate feasibility, acceptability, and usability, giving professionals time to adapt to the new work processes. Additionally, by stepping back and forth between the development phase and feasibility phase [18], work processes were discussed, evaluated, and adjusted during monthly working group meetings.

The feasibility phase concluded with a constructive final evaluation in an interactive co-creation meeting [20] with three patient representatives and eleven healthcare professionals. In this meeting, we summarised HBGR trajectory version 1.5, including the feedback and experiences of patients, patient representatives, and healthcare professionals gathered during the pilot. This was discussed in subgroups before being presented to the entire group. Ultimately, the group agreed on the final design and content of HBGR trajectory version 2.0.

We recorded all the semi-structured interviews and co-creation sessions on tape and transcribed them verbatim. The research and personal data were stored in a secure folder within Amsterdam UMC and pseudonymised in the transcript. Field notes were made throughout the entire process and entered into an Excel database, along with observational data regarding the progress of the individual trajectories. Notes and action plans were made and stored in a shared group file during each working group meeting. AP was responsible for maintaining this file.

### Data analysis

We employed a hybrid approach to analyse the qualitative data from patient interviews and co-creation meetings, integrating both deductive and inductive methods, as described by Bingham [23]. To ensure alignment with established research frameworks initially, AP employed a deductive analysis to categorise data based on knowledge from previous research [10, 11, 24], a structured classification of the main topics was made: preparing for discharge home, transition to home, daily functioning before, during and after HBGR, location of treatment use of eHealth, informal care, CC nursing, finishing of HBGR, and patient and professionals experience with HBGR. Subsequently, AP conducted an inductive analysis to identify emerging (sub) themes and patterns from the data [23, 25]. After the initial analysis, the results were thoroughly discussed with the research team to reach a consensus. This flexible methodology facilitates the recognition and integration of new insights derived directly from the data, thereby achieving both theoretical relevance and novel findings.

The analysis of patients’ and professionals’ experiences was used as input for any necessary adjustments to the HBGR trajectory during the development and feasibility phase. The working group meetings, including field notes, were used as additional input during the analysis, which followed a plan-do-check-act cycle [26].

### Logic model

To enhance understanding and provide a structured summary of the HBGR trajectory redesign process, we organised all research steps into a streamlined framework, starting with the contextual analysis and concluding with its impact on the professional field. This framework is known as a logic model and is part of the MRC framework [27].

### Ethics approval and consent to participate

This study was approved by the Medical Ethics Committee of the University of Amsterdam in the Netherlands (protocol ID 2023.0254). All participants provided informed consent prior to participating in this study.

## Results

### Characteristics participants

Table 1, shows the characteristics of the three groups of participants, and Fig. 2 show the patient participation flowchart. The ten included patients had a median age of 79 (IQR 71-81.5), of which seven (70%) had a stroke diagnosis, one had an orthopaedic diagnosis, one had an oncologic diagnosis, and one had general malaise. Reasons to not follow HBGR were: discharge to long-term care (*n* = 10), one discipline needed (*n* = 9), no rehabilitation goals (*n* = 7), death (*n* = 2), and hospital re-admission (*n* = 1). The median duration of IGR was 36.5 days (IQR 19-38.5), shorter than the national median of 43 days [28]. For HBGR, it was 36 days (IQR 21.5–45). The study involved the participation of four patient representatives, including two former patients and two informal caregivers. Eleven healthcare professionals from ten different disciplines participated in the co-creation sessions, all experienced with HBGR, with 90% being women.

**Table 1:** Characteristics of the three groups of participants

***Patient participants***						
**Participant**	**Gender**	**Age**	**Marital status**	**Diagnosis (multi-morbidity)**	Length of IGR* (days)	Length of HBGR**(days)
1	Male	82	Living with partner	Stroke	39	49
2	Male	77	Living with partner	Stroke	17	15
3	Male	79	Living with partner	Stroke	37	36
4	Female	86	Living alone	Stroke	36	36
5	Male	64	Living with partner	Oncology	40	23
6	Female	68	Living with partner	Total knee replacement	37	66
7	Male	69	Living with partner	Stroke/Conversion syndrome	19	45
8	Female	80	Living with partner	Stroke	45	45
9	Female	86	Widow, living alone	General malaise	19	21
10	Female	79	Living with partner	Stroke	16	17
*Median* *(IQR)*		79 (71-81.5)			36,5 (19.5–38.5)	36 (21.5–45)
***Patient representative participants***						
**Participant**	**Gender**		**Role**	**Experience HBGR**		
1	Male		Former patient	Yes		
2	Male		Former patient	Yes		
3	Male		Caregiver	Yes		
4	Female		Caregiver	Yes		
***Healthcare professional participants***						
**Participant**	**Gender**		**Discipline**	**Experience HBGR**		
1^a^	Female		Manager GR department/elderly care physician	Yes		
2^a^	Female		Coordinator GR/physiotherapist	Yes		
3^a^	Female		Community care nurse	Yes		
4^a^	Female		GR nurse	Yes		
5^a^	Female		Occupational therapist	Yes		
6	Female		Dietician	Yes		
7	Female		Psychologist	Yes		
8^a^	Male		Physiotherapist	Yes		
9^a^	Female		Nurse practitioner	Yes		
10	Female		Social worker	Yes		
11	Female		Speech therapist	Yes		

*IGR = inpatient geriatric rehabilitation, **HBGR = home-based geriatric rehabilitation, ^a^*Working group member*

**Fig. 3:**
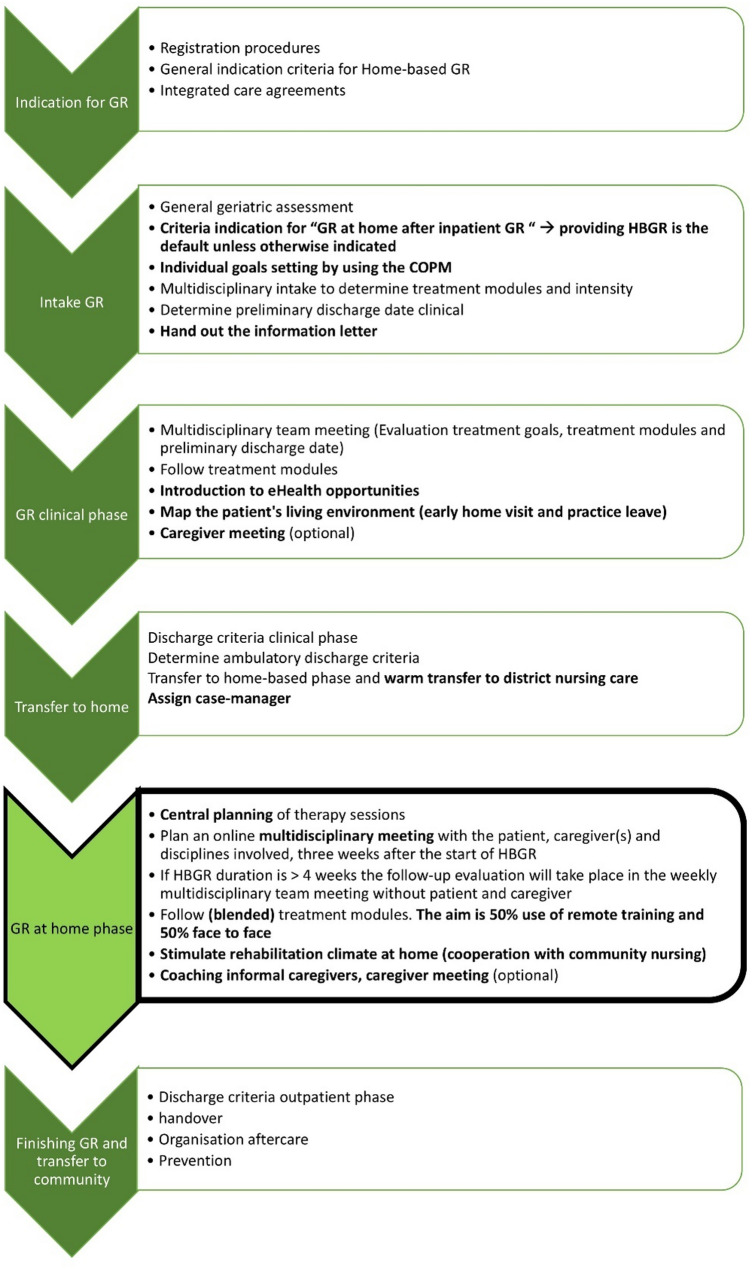
Home-based geriatric rehabilitation trajectory and inpatient preparation, version 2.0. *COPM = Canadian Occupational Performance Measure, HBGR = Home-Based Geriatric Rehabilitation*

### Redesigning process

#### Development phase

During the development phase, the initial HBGR trajectory version 1.0 was refined into version 1.5 in co-creation with professionals and patient representatives. Forty topics were gathered and discussed, divided into the building blocks, structure, process, environment, and outcome (see Appendix 3). Of these topics, eleven elements were identified as essential to developing, refining, and adding to the HBGR trajectory. The order in which the elements would be implemented during the feasibility phase was prioritised. First, the elements that needed no or limited adaptation were individualised goal setting, providing HBGR is the default unless otherwise indicated, information letter about HBGR, early mapping of the patient’s living environment, central planning, collaboration with CC nursing concerning the warm transfer, and providing therapy in the home setting. Secondly, the elements needing average development or refinement were the rehabilitation coordinator, education for CC nursing concerning rehabilitation at home, online evaluation meetings, and education meetings for informal caregivers. Finally, the blended use of eHealth (exercise apps and sensor technology) was implemented, which required the most development on different levels, such as arranging the equipment and adapting the working process.

#### Feasibility phase

Finally, the existing HBGR trajectory version 1.0 has been redesigned throughout this study into version 2.0, as demonstrated in Fig. 3. The eleven key elements are highlighted in bold and briefly defined in Appendix 4.

**Fig. 4:**
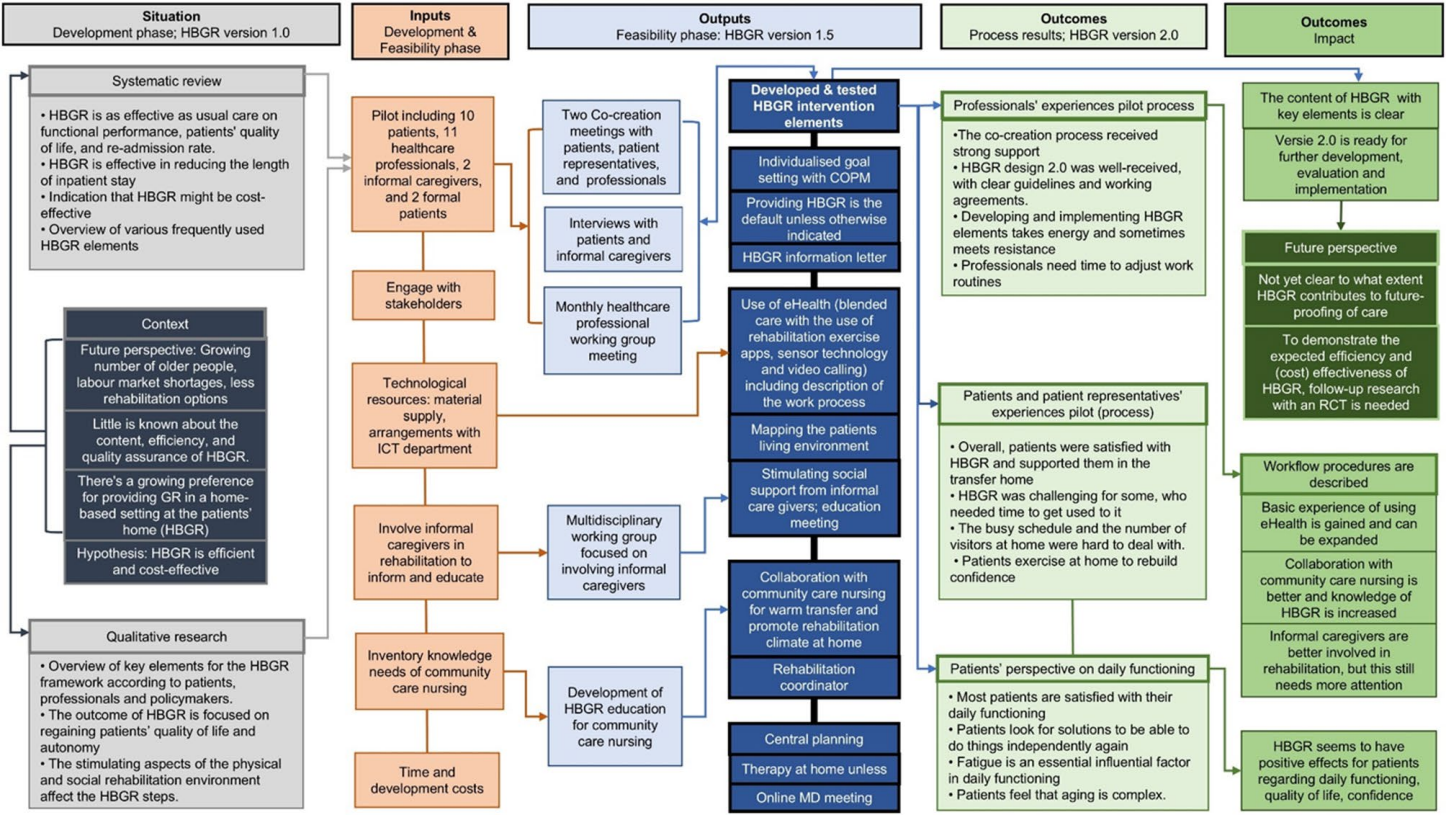
Logic model redesign Home-based GR trajectory. The situation box reflects the context, knowledge, needs and priorities for this study. What is invested in the pilot is shown in the box input. The output box reflects the participation and activities performed during the pilot. Finally the two outcomes boxes show the short term pilot process results, and the long term impact for the future. *COPM = Canadian Occupational Performance Measure, HBGR = Home-Based Geriatric Rehabilitation, MD meeting = Multidisciplinary Meeting*

##### HBGR trajectory version 2.0

The HBGR workflow procedures were described throughout this study, where three elements showed the most remarkable development.

Firstly, the essential experience in using eHealth is gained and can be expanded. Due to the late delivery of materials, the technological tools were used for a short time and applied to only five patients. Valuable experiences were gained by integrating remote training using an exercise app with face-to-face treatment (blended use of eHealth). Both patients and professionals indicated positive experiences like stimulating patients to do exercises, promoting patients’ autonomy, and saving time.

Furthermore, introducing technology early in the inpatient setting seemed essential since it gave the patients enough time to learn how it works. However, difficulties were encountered in adapting to the daily routine. Professionals have stressed the importance of having enough time and space for planning to gain valuable experiences for the future and adjust practices as needed. A clear outline of the blended working process, education plan, and IT support is also necessary.

Secondly, collaboration with CC nurses is improved, and their knowledge of HBGR is increased. The pilot project aimed to enhance cooperation with stakeholders involved in HBGR. The co-creation meetings concluded that CC nurses play a pivotal role in this process, and involving them in the rehabilitation trajectory at an early stage is crucial. The feedback from professionals also highlighted the need to enhance CC nurses’ understanding of the home rehabilitation environment to facilitate a smooth transition. A post-training survey revealed that the training was well-received, and the knowledge gained can be directly applied in daily practice. Efforts have been made to establish connections with CC nursing, and it is now imperative to strengthen this collaboration and ensure its continuity.

Finally, informal caregivers are better involved in rehabilitation, but this still needs more attention. The informal caregivers who attended the caregivers’ information meeting felt well-informed. However, some noted that it needs to be clarified when the meetings are scheduled, and that communication could be improved. They mentioned that it is beneficial to observe therapy sessions, enabling them to ask questions, stay informed about their loved one’s progress, and learn which exercises can be done at home. However, it was also mentioned that not every caregiver may need this, for instance, if they already feel burdened by their care responsibilities.

#### Experiences and perspectives

During the feasibility phase, the experiences and perspectives of the patients, patient representatives, and professionals regarding the redesigning process, pilot testing, and the final HBGR version 2.0 were gathered.

### Patients and patient representatives

Overall, patients were satisfied with HBGR and recognized the added value and usability, as it supported them in transitioning home. They reported being satisfied with the entire process, including the rehabilitation at home. Also, caregivers see the added value: “*Of course*,* this is completely focused on self-reliance and if I see how my mother manages by herself. She says*,* without the therapists (HBGR)*,* I definitely wouldn’t have been able to do this…. It is more focused on the home situation and the everyday situation. I think it is perfect.” (Caregiver)* The patients mentioned various elements: (i) Getting to know the CC nurse who takes over the care at home beforehand gives them confidence, making the transition less challenging. (ii) Patients appreciated therapists visiting them at home for rehabilitation, allowing them to practice in their environment and schedule multiple therapies on the same day. (iii) Most patients did not notice a rehabilitation coordinator was appointed during the HBGR trajectory and was seen as a regular part of the rehabilitation process Iv) Patients found the Multidisciplinary evaluation (MDE) meeting valuable, especially when discussing rehabilitation goals. However, they noted that the meeting provided less or no added value if the trajectory was clear.

HBGR was challenging for some patients who needed time to get used to it. Patients found comfort in returning home to a familiar environment where they could do things at their own pace without feeling constantly observed. This feeling was mainly expressed by patients with a partner assisting with household tasks and self-care. Patients without a partner also described being back home as lovely but often encountered challenges. They mentioned that it took time to readjust to doing everything independently again at home, as many responsibilities had been taken over during their inpatient stay: *“At first*,* it was a bit disappointing. You’re thrown back on yourself*,* and now you have to do everything yourself. You come home*,* and so you have to work*,* so to speak.” (Interview 6)* Additionally, patients highlighted the complexities of ageing, expressing frustration at the gradual loss of abilities, which made everyday activities more difficult.

The busy schedule and the number of visitors at home were hard to deal with. Many patients report feeling overwhelmed during the HBGR due to a high volume of visitors, a packed therapy schedule, and numerous doctor’s appointments: *“Well*,* you know*,* you have to be left alone at some point.” (Interview 2)* Coping with the resulting fatigue is challenging. Fatigue is a common complaint indicated by patients. It impacts their daily functioning and prevents them from operating at their desired level. Many patients indicate they keep looking for solutions to do things independently again; *“I just keep going. I have said I will*,* and I shall*,* and I must.” (Interview 4)* As a result, most patients are satisfied with their daily functioning and experience that the progress is still visible or that they are functioning at their old level again. They indicated that the home rehabilitation phase is acceptable since it helped them regain autonomy partly because they could exercise practically in their home environment.

Patients indicated that they rebuild confidence through guidance during the HBGR phase and by doing homework exercises at home. However, some patients are more motivated to exercise than others. Homework programs were delivered either on paper or through an exercise app. The use of technology was well received by patients, as an exercise app with a personalised exercise program encouraged them to start or continue exercising independently. Due to an introduction during the inpatient period, the patient felt more confident working with the technology. The exercise app also facilitated video calling and remote treatment, which worked well for them in combination with face-to-face therapy: *“They came to my house once a week to do the exercises. I didn’t feel that that was too little*,* and I liked that video call with the physiotherapist to see how far along I am and what they think of it. And so that also gives a bit of a boost.” (Interview 8)* Informal caregivers also participated in carrying out homework exercises to different extents. Some caregivers were closely involved and willing to assist with the exercise program. At the same time, others wanted to maintain distance to avoid becoming too involved with the care and preserve their partner’s role.

### Healthcare professionals

The co-creation process received strong support from all participants. The professionals positively received the iterative development process as it allowed for immediate testing and necessary adjustments. However, some professionals highlighted that all colleagues not involved with the working group needed to be more informed: *“The entire process description of the outpatient trajectory has not yet been widely shared*,* but it should be so that it is clear to everyone what the agreements are. In this way*,* we and the entire treatment team can also better manage outpatient rehabilitation and work towards reducing the length of inpatient stay.” (Elderly care physician)*.

HBGR Design 1.5 received positive feedback from professionals, who found it feasible and usable, particularly when offering clear guidelines and working agreements. These agreements create awareness and clarify that a larger group of patients is eligible for HBGR than previously thought. The professionals acknowledge that they must learn to have faith that a patient can go home safely with an HBGR trajectory: “*It is excellent that there is now an outpatient trajectory description and that we also identify sooner when someone can go home because we have made arrangements on who is eligible for HBGR*,* however*,* sometimes we are still too cautious about that*.” *(Occupational therapist)* Furthermore, collaboration with CC nursing was beneficial as it facilitated a smooth patient transition home, with frequent patient interaction and comprehensive overviews during MDE meetings. The professionals also provided positive feedback about the home rehabilitation training provided to CC nurses, noting that it was practical and immediately applicable to daily routines. Additionally, the professionals welcomed the introduction of a rehabilitation coordinator (also known as a case manager), attributing the positive impact to the coordinator’s role in ensuring a seamless process and serving as a point of contact for patients, which led to more efficient care.

Besides the positive experiences, the working group members indicated that developing and implementing HBGR elements takes energy and sometimes meets resistance. The group members perceived resistance at various points, for example, with the introduction of the MDE meeting. This evaluation meeting previously had a different format that did not satisfy both patients and professionals, so both groups were not motivated to participate. The physicians initially resisted the newly introduced MDE meeting, fearing it would be time-consuming and add little value to the rehabilitation process. However, they now see it as beneficial, providing timely direction for the rehabilitation plan and better coordination among the treatment team members. Although a nurse practitioner mentioned: “*The MDE meeting is not always perceived as valuable. Better agreements need to be made on when to schedule it and when not to.” (Nurse practitioner)*.

Furthermore, the professionals indicated that they need time to adjust work routines; they want to, but they often do not know how due to lack of time. They experienced that implementing eHealth applications requires extra time and effort because adapting to remote treatment, transitioning to a coaching role, and guiding patients from a distance was challenging. According to them, a transparent working process can help implement the new procedure: *“It is important to have a work process description for working with iPads so that this becomes fixed in the daily structure. Then*,* it will also be easier for everyone to start working with them.” (Physiotherapist)* Professionals observed that older individuals can use technology but often need more guidance. They noted the benefits of technology, mentioning that it saves time, gives patients more autonomy, and adds to self-management while reducing travel time.

#### Logic model

Figure 4 presents the structured framework that summarises the redesign process of the HBGR trajectory. It contains four components: [1] the situation, which reflects the context, knowledge, needs, and priorities of this study; [2] the input, representing the investments made in the pilot; [3] the output, which captures participation and activities performed during the pilot; and [4] the outcome, presenting both the short-term results of the pilot process and its long-term impact on the future. This model provides a comprehensive overview of the research steps, illustrating the sequential progression from contextual analysis to the anticipated impact on the professional field.”

## Discussion

With this redesign and feasibility study, using the MRC framework, the HBGR trajectory is redesigned and tested in co-creation with patients, patient representatives and healthcare professionals, leading to HGBR trajectory version 2.0. This trajectory is found to be feasible and holds substantial positive value in terms of acceptability and usability for patients, their informal caregivers, patient representatives, and healthcare professionals. It is pioneering within the field of geriatric rehabilitation in the Netherlands that three stakeholder groups have been closely involved in the redesign process. This aligns with international efforts to promote patient-centred care through co-creation. Studies from the UK and Scandinavia show that involving patients and healthcare professionals in an iterative process leads to more sustainable rehabilitation pathways [12, 13, 29–31]. While the studies of Röpke [13] and Kristensen [30] are concentrated on single-diagnosis pathways, our methodology distinguishes itself by encompassing a wider range of diagnostic groups, thereby making it applicable across various healthcare systems. Our study resulted in a clear and practical overview of the content of the HBGR trajectory, providing an outline of eleven organisational elements with described working processes. The various elements underwent iterative development, evaluation, and adjustment cycles to optimise usability and acceptability, promising substantial patient benefits. The eleven elements represent a selection of 40 named development topics from the initial co-creation meeting, suggesting that many opportunities and developments remain. Nevertheless, this pilot study was an essential step in the structured application of HBGR, offering new insights and several important lessons.

Firstly, the gained experiences in using technology pointed out the importance of introducing technological tools during the inpatient GR. This method allows people to learn how to operate the technology and use it optimally during the HBGR trajectory in the home environment [32, 33]. Professionals should consider the barriers and facilitators that affect older adults’ use of technology [34], such as the need for more time to learn [35]. Therefore, we introduced a digital exercise group in the pilot where patients received an explanation about the technology to be used, went through the exercise programme and practised video calling. This regular coaching and support helped them to gain confidence and stimulated the joy of using it.

It can also increase their motivation to exercise and improve their quality of life [36, 37]. Research from Canada and Australia shows that effective technology use can improve outcomes for older adults [38, 39]. Similar to the study of Kraaijkamp et al. [24], the integration of technological tools in daily practice (blended care) presented challenges as professionals had to adapt to new technology and a different way of working, which required time and effort [40]. For example, remote training made it difficult for professionals to switch to a coaching rather than a treatment role where they think they can provide less supervision [40, 41]. During this study, a start was made with blended working [34, 40, 41], matching the global shift toward digital rehabilitation. However, more experience is needed to describe the most optimal way of blended working, taking into account scientific studies.

Secondly, we learned more about the crucial role of informal caregivers in home rehabilitation [42, 43]. The informal caregivers felt more supported and well-informed due to the information meetings about the rehabilitation trajectory at home and the opportunity to participate in therapy sessions. Conversely, some caregivers indicated that being involved in the therapy sessions can be stressful since it changes the relationship with their partner [44]. This aligns with other international studies, which demonstrate that caregivers often feel overwhelmed due to the new responsibilities [29, 30] and the risks associated with caregiver burden [45, 46]. Therefore, it is beneficial for them to be recognised as stakeholders, be well-informed, and be involved in rehabilitation [42–44]. Besides the caregivers, the CC nurses play a crucial role in HBGR. Through this study, the collaboration with CC nursing and their knowledge about rehabilitation at home is improved. This improvement could positively impact the smooth transfer home and rehabilitation climate at home [43, 47, 48]. From the multidisciplinary team, the CC nurses see the patient and their caregivers most frequently, where they can stimulate the patient to exercise and support caregivers in addition to their care tasks [47]. Our study results can be seen as a first step towards implementing integrated care and enhancing collaboration between different settings to improve care quality [49, 50].

Finally, we learned that engaging and working with diverse stakeholders of the HBGR trajectory is crucial in redesigning a well-supported HBGR trajectory. This approach ensured that knowledge was shared effectively with a broad group of stakeholders outside the working group and kept them informed of developments [18, 51, 52]. Stakeholder engagement can strengthen collaboration in an integrated care chain [51, 52] and increase the likelihood of successful implementation [18, 51]. Moreover, including the experiences and opinions of (former) patients and informal caregivers in the redesigning process is vital, as the patient should be at the centre of the rehabilitation process [24, 51, 53]. However, finding participants for this role can be challenging, as many may struggle to express their opinions effectively or lack prior experience in the rehabilitation process, making it difficult for them to provide meaningful input.

From a future perspective, the study highlights critical insights for evaluating and implementing the HBGR design following the MRC framework [18]. Nevertheless, the analysis revealed that the total GR trajectory including HBGR was lengthy and should be shortened to address societal challenges. However, the small participant size in this pilot may have skewed the trajectory duration due to outliers. Additional research is needed for a more accurate understanding. Furthermore, resistance was observed among professionals when introducing new HBGR elements. This suggests that integrating HBGR 2.0 into standard procedures necessitates a phased implementation process, considering the challenges and resistance inherent in change [54]. Professionals require adequate guidance, time, knowledge, and motivation to minimise stress levels and facilitate adaptation to the new situation. A practical implementation strategy is recommended to ensure the success of this change process and enhance care quality [54, 55].

Conducting follow-up research is crucial to demonstrating the expected efficiency and (cost) effectiveness of HBGR, serving as a basis for its implementation in daily practice if proven effective [18], while also clarifying its contribution to the futureproofing of GR [5], which is influenced by factors such as costs, technological resources, and professionals’ knowledge and skills. A more extensive feasibility study—including additional standardised assessments—would provide further insights into the intervention’s effectiveness, making this an important consideration for future research and subsequent evaluation phases of HBGR implementation.

### Strengths and limitations

The strength of this research is its action-oriented iterative approach [51, 52], in which development and implementation were carried out simultaneously in collaboration with various stakeholder groups [33]. This method may increase its acceptability and usability in daily practice as also demonstrated by Tembo et al. [18, 51].

Another strength is that the development process of HBGR trajectory version 2.0, including all the different steps, has been simplistically mapped through a logic model. This model allowed for proper connections and provided an overview of components that required attention during the process.

Moreover, the study results directly apply to daily practice since they provide a detailed description of the trajectory content, including work procedures.

The limitation of this study is the small sample size, with 70% of the included patients diagnosed with stroke. This may affect the external validity of the findings [56, 57]. On the other hand, the pilot took place in a setting whose target group is like that in different settings [3, 48]. We acknowledge that the GR trajectory 2.0 has been developed for a Dutch situation. However, we used the GR key elements where international consensus has already been reached by the Delphi study of van Balen et al. [3].

## Conclusion

With a redesigning and feasibility study, the HBGR trajectory is redesigned into an optimal version 2.0. The redesigned trajectory was well-received by patients, patient representatives, and healthcare professionals, who found it feasible, acceptable and usable. Eleven elements were added to the HBGR trajectory: individualised goal setting, providing HBGR is the default unless otherwise indicated, information letter, blended eHealth, mapping patient living environment, stimulation support from informal caregivers, collaboration with CC nursing, rehabilitation coordinator, central planning, therapy at home, online multidisciplinary evaluation. The approach aligns with global trends in patient-centred rehabilitation, blended care, and the involvement of informal caregivers. The study showed that involving (former) patients, informal caregivers, and healthcare professionals was critical in promoting successful development and gaining support. The HBGR trajectory 2.0 is ready for evaluation and implementation in follow-up effectiveness research.

## Electronic supplementary material

Below is the link to the electronic supplementary material.

Supplementary Material 1

Supplementary Material 2

Supplementary Material 3

Supplementary Material 4

## Data Availability

The datasets used and/or analysed during the current study are available from the corresponding author on reasonable request.

## Abbreviations

CC nurse/nursing: Community Care nurse/nursing
COPM: Canadian Occupational Performance Measure
GR: Geriatric Rehabilitation
HBGR: Home-Based Geriatric Rehabilitation
IGR: Inpatient Geriatric Rehabilitation
MD meeting: Multidisciplinary meeting
MDE meeting: Multidisciplinary Evaluation meeting
MRC framework: Medical Research Council framework
SNF: Skilled Nursing Facility
IQR: Interquartile Range
