# Supplementary material for: Home-based geriatric rehabilitation after inpatient rehabilitation: a redesign and feasibility study
Source: BMC Geriatr. 2025 Jun 2;25:398. doi: 10.1186/s12877-025-06043-z (PMC12128391; doi:10.1186/s12877-025-06043-z)
Supplement: Supplementary file 1 — Supplementary Material 1 [file 12877_2025_6043_MOESM1_ESM.pdf]

Appendix 1 Patient interviews

Interview guide for the semi-structured patient interviews

Thank you for taking the time to have this conversation. We are here together to discuss your home rehabilitation. We have redesigned the home rehabilitation trajectory and added a number of elements. We are testing this in practice and are very curious about how you have experienced it. We would like to learn from your experiences to further shape and develop this form of care. Let me first introduce myself.<br><br>**Begin.** The interview will last about 45-60 minutes. We would like to record this interview so that we can listen to it later for our research; of course, it will remain completely anonymous and be deleted after this research. Your answers will be kept for 10 years. We want you to know that there are no wrong answers during this interview and that any information you give us will remain within these walls, so feel free to say anything you want. Do you have any questions for us in advance? **Start recording.**			
**Introduction**	Experience	How did you experience home rehabilitation?	Could you tell us a bit more about your experience of rehabilitation at home?
**Outcomes**			
	Transition home	How did you experience the transition from the rehabilitation ward to home? (exciting, emotions)	
		Was this the right time for you to start rehabilitating at home?	In hindsight, would you have liked to go home faster or later?
			Can you explain this?
		How are you prepared for going home?	
	Daily functioning (activities, quality of life)	How has home rehabilitation helped you carry out your daily activities? (e.g. hobbies, shopping, housekeeping, self-care, quality of life)	What were the good points and not so good points

			How would you like to see these improved?
**Process**			
	Practice leave	How did you experience the practice leave?	Can you tell a bit more about it? (time of day, experience, were you alone, form filled in, did it feel like good preparation, what was the timing)
	Information letter	In what way were you provided with information (written or oral) about the home rehabilitation trajectory	To what extent was this information sufficient?
			Advice for improvement?
	Treatment	Can you describe what the treatment has been like for you since returning home?	Content, frequency, professionals, attitude, collaboration, intensity, duration
		Did you have the same therapists at home as during the admission?	Advantages and disadvantages?
		Where does your therapy take place?	On location, how did you arrange transportation? And is this to your liking?
		Do you also get homework exercises?	In what form?
		Advice for improvement	
	EHealth, blended care	For which treatment components did you use the iPad? (therapy, homework, remote training, MD conversation)	How did it work? What is your experience with it?
			In what other ways could it be used?
		Instead of face-to-face contact?	How did that work? What is your experience with this?
		Advice for improvement?	
	Rehabilitation Coordinator	Was there a rehabilitation coordinator during your trajectory?	If yes, how were you informed about the coordinator? What was

			the contact like? Can you tell me more about that?
			If not, did you miss it and why?
		Advice for improvement?	
	Community care nursing	When was your first contact with the district nurse? (Digital, location or at home)	How was that for you?
		How was the transition of care from clinical to home?	Can you tell a bit more about that?
		To what extent does community care nursing encourage rehabilitation at home	In what way? What is your experience with this?
		Advice for improvement?	
	MD meeting	Three weeks after discharge, there was an MD meeting with all disciplines involved, such as physio, care, and specialist elderly care. How did you experience that?	Added value? Was it online or on location? What was pleasant/unpleasant.
		Advice for improvement?	
	Central planning	How did the appointment planning go?	What was your experience? Advice for improvement?
	Informal caregiver meeting	How did the appointment planning go?	How could it have been otherwise?
		What impact has the informal caregivers meeting had, or could it have on the level of the caregiver's burden?	
**Structure**			
	iPad/technology	What technological tools were used during your HBGR	How did that work for you? What was the added value of this?
			To what extent did you possess these technological tools yourself?

	Collaboration therapists with community care nursing teams	How is cooperation with rehabilitation therapists and community care?	
		Do you notice that you miss contact hours with therapists at home?	
	Development Home Rehabilitation Trajectory	How did you experience the newly developed home rehabilitation pathway?	Do you have any additions/improvements to this?
		What is the added value of HBGR?	Can you explain this?
**Additional information**			

Closure

Do you have any questions for me?

I would like to thank you very much for this conversation and the time you have given us. Your contribution will lead to a beautiful article and a valuable addition to this form of care.

Interview analysis, codes

Main themes	Subthemes	subtopics
**Experience HBGR**	Difficult and heavy	Busy agenda
	Satisfied and added value	
	Feeling hard to put into words	
**Inpatient period**	Experience	Helped in recovery
		You didn't have to do anything
		Clinically different from home
	Functioning during the inpatient period	Mobility possibilities
		It didn't function properly yet
		Already functioned quite well clinically
	Building/environment	
	Care	

**Functioning before GR**		
**Functioning during HBGR**	Stabilisation in the level of functioning	
	Building up functioning	Building trust
		Practice and exercise at home
		What I had to do, I did
		At home, you will do more and pick up activities
	Functioning when returning home	insecure
		Functioning was not optimal but good enough to go home
**Functioning after HBGR**	Aftercare	
	What I can do myself, I do myself	
	Fatigue	
	Functioning does not go as desired	
	Satisfied with performance	
	Participation: take up activities and tasks	
**Technology Usage**	Received exercise program on the computer	
	Video calling	
	Hipper used for alignment of load-load capacity	
	It could be complicated with the older people	
	Not handy with phones and technology	
	Can handle computers well	
	No technology deployed during HBGR	
**Preparing for home**	Plenty of time to prepare	
	Home modifications	
	About HBGR	
	Practice leave	

**Transition home and timing**	Nice to be home	
	Partner at home makes it easier	
	It's just different at home	
	It wasn't easy to go home	strange
		afraid
		alone
		You have to do everything yourself
		Disappointing
	I wanted to go home myself	
	Timing was right	
**Informal care**	Help from partner	Is insecure
		Overprotective
		Takes over tasks
		Helps or stimulates doing training exercises
		Heavy for partner
	The family provides support and additional explanations	Gives support
		Made the discharge home possible
		Patients don't want to ask for help; children are busy and have their own life
	Attendance at therapy	Gives information and guidance how to help the patient
	Caregiver Meeting	Added value
**MD evaluation meeting – conclusion HBGR**	Didn't have a meeting	Not clear what HBGR is and what the difference is with primary care
	Did have a meeting	Good experience
		Online; saved time and works well
		No obvious addition

		On location: advance to meet other people, however costs more time
**Rehabilitation Coordinator**	was not clear if a coordinator was present	Was missed by the patient and caregiver
	Positive feedback about the coordinator	Provides support if you can reach someone
		Was very knowledgeable and embraced the entire care process
**Community care nursing**	Warm transfer	Nice to meet the community nurse in front
	Experience	Satisfied with the care
		Nice that they are there to help you
		Don't want to be dependent
		Many different nurses
**Treatment plan**	Being able to set your own goals	
	No insight into the plan	I'm not interested in that
**Location treatment**	No preference	
	At home	practical
		No materials missed
		positive
	Practice on location	More possibilities
		Feels more pressure to do something
		At own request, want to get out of the house, want to be in charge of the day planning
	combination	advantages of both places
**Therapy in disciplines**	Satisfied with the difficulty of exercises	

	Tailored to the patient	
	Therapists work together	
**Central planning**	Worked well	could indicate wishes
