# Supplementary material for: Home-based geriatric rehabilitation after inpatient rehabilitation: a redesign and feasibility study
Source: BMC Geriatr. 2025 Jun 2;25:398. doi: 10.1186/s12877-025-06043-z (PMC12128391; doi:10.1186/s12877-025-06043-z)
Supplement: Supplementary file 2 — Supplementary Material 2 [file 12877_2025_6043_MOESM2_ESM.pdf]

Appendix 2 Pilot HBGR organisation and meetings

Table Pilot organisation and meetings

Activity	Aim	Participants	Results
Preparation pilot	Writing the protocol and recruiting healthcare professionals and patient representatives	Research team: AP, MH, MP, BB	The study protocol is ready and approved by the Medical Ethics Committee of the University of Amsterdam in The Netherlands (protocol ID 2023.0254)<br>The participants for the working group and co-creation meetings are recruited
Kick off pilot working group	First meeting with the working group to make work agreements, division of tasks, and discuss the study planning	-Working group members (n=7): a physiotherapist, an occupational therapist, an elderly care physician, a nurse specialist, a Geriatric rehabilitation nurse, a community care nurse, the Manager of the GR department. - Research team member AP, and head of the scientific research department.	The planning of the pilot is clear.<br>There is clarity on the division of tasks for example, chairman, coordinator of meetings, and note-taker.<br>Agreements have been made as to who has which HBGR element as its area of focus.
Kick off pilot	To make the geriatric rehabilitation department aware of the pilot.<br>To make work appointments	All stakeholders of the department: healthcare professionals, management, working group members	Through a presentation at an information meeting the stakeholders are informed about the pilots planning, content and goals.
Co-creation meeting 1	Interactive meeting to redesign the existing Home-based GR trajectory version 1.0 and to shape the preconditions for an optimally organised trajectory.	- Working group members (n=7)<br>- Former patient 1 (n=1),<br>- Additional healthcare professionals (n=4): speech therapist, dietician, psychologist, social worker,	The Home-based GR trajectory version 1.5 is designed based on previous studies and two guiding principles:<br>- the patient journey (which journey the patient goes through schematically)<br>- the structure, process, outcome model of Jesus et al., 2015)

		- Research member of the national living lab "Better@home" (n=1)	Division of tasks between the working group members; agreements on what focus each working group member has
Workgroup meeting 1-5	Monthly meetings to monitor, evaluate, and adjust the developments of the HBGR elements to be introduced where necessary. In doing so, make follow-up agreements and monitor whether they are met. What are the impeding and stimulating factors, and how do we act upon them?	Working group members (n=7)	Each working group meeting an overview was made of what is going well in practice and which elements need attention and/or adjustments.<br>It was agreed on which work processes should be described by the next meeting and by whom.<br>The points for attention were the evaluation multidisciplinary meeting, the technological resources that are not yet available, the provision of information to the patient and their informal caregivers, training for community care nursing, warm transfer to community care nursing, case management.<br>The patients' experiences were included in these interim evaluations and adjustments of the Home-based trajectory.
Co-creation meeting 2	Interactive meeting to reach final agreement on Home-based GR trajectory design version 2.0.	- Working group members (n=7)<br>- former patient 2 (n=1),<br>- informal caregiver 1 and 2 (n=2) ,<br>- Additional healthcare professionals (n=4): speech therapist, dietician, psychologist, social worker,<br>- Research member of the national living lab "Better@home" (n=1)	In this meeting a summarise was given of the, a summary of the home-based GR trajectory version 1.5 was given based on the feedback and experiences of patients, patient representatives, and healthcare professionals gained during the pilot. This was discussed in subgroups and then with the entire group. Finally, Home-based GR trajectory version 1.5 based on the feedback and experiences of patients, patient representatives, and healthcare professionals gained during the pilot. This was discussed in subgroups and then with the entire group. Finally, agreement was reached on the final design and content of Home-based GR trajectory version 2.0.
