# Supplementary figures and images for: Home-based geriatric rehabilitation after inpatient rehabilitation: a redesign and feasibility study
Source: BMC Geriatr. 2025 Jun 2;25:398. doi: 10.1186/s12877-025-06043-z (PMC12128391; doi:10.1186/s12877-025-06043-z)

## Appendix 3 Home-based GR trajectory version 1.0

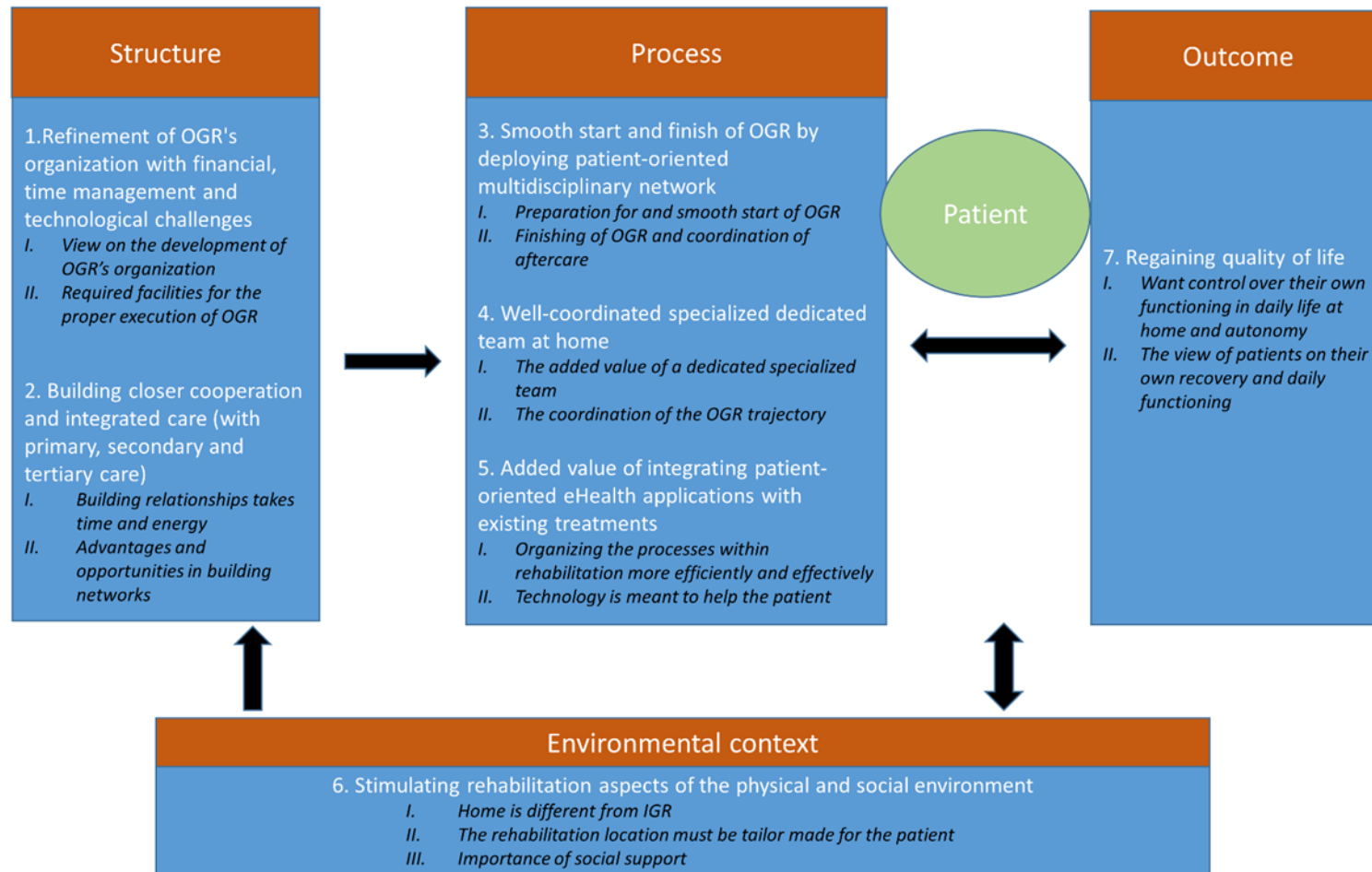

Supplement: Supplementary file 3 — Supplementary Material 3 [file 12877_2025_6043_MOESM3_ESM.pdf]
