# Supplementary material for: Home-based geriatric rehabilitation after inpatient rehabilitation: a redesign and feasibility study
Source: BMC Geriatr. 2025 Jun 2;25:398. doi: 10.1186/s12877-025-06043-z (PMC12128391; doi:10.1186/s12877-025-06043-z)
Supplement: Supplementary file 4 — Supplementary Material 4 [file 12877_2025_6043_MOESM4_ESM.pdf]

Appendix 4 Home-based GR (HBGR)trajectory key elements

The HBGR trajectory version 2.0 contains eleven essential elements, briefly discussed below.

1. Individualized goal setting with Canadian Occupational Performance Measure (COPM)
"The COPM is a patient-centred, occupation-focused outcome measure that detects changes in perceived daily performance over time. The COPM consist of two subscales: the "performance" subscale (COPM-p) and the "satisfaction" subscale (COPM-s). Through a semi-structured interview, patients prioritize up to five daily activities they consider most important and would like to improve. The patients subsequently rate these activities on a 10-point scale regarding performance (COPM-p) and satisfaction (COPM-s) ranging from 1 to 10" (1). It is an essential tool used by occupational therapists to set treatment goals based on a patient's daily activity difficulties. Informal caregivers may provide input if the patient can't identify their challenges. The identified problems are integrated into the treatment plan, and all disciplines contribute their own goals and actions. Scores are used to gauge the patient's perception of their actions, and the COPM is repeated at the end of inpatient and outpatient care to measure changes. The outcomes guide follow-up treatments and are discussed in medical team meetings.

2. Instruction always HBGR unless….
The instruction states that HBGR is a standard part of the overall GR pathway. Every patient is eligible for HBGR unless one of the following reasons applies: (i) rehabilitation goals have been achieved, (ii) multidisciplinary treatment is no longer necessary, (iii) the patient refuses or lacks motivation, (iv) the patient lives outside the region (>15 minutes driving distance from the skilled nursing facility/rehabilitation ward) and does not have the means to come to the clinic for treatment.

3. HBGR information letter
The letter to be handed over on the day of Inpatient GR admission contains information about what an OGR trajectory entails and what the patient and caregiver(s) can expect.

4. Use of E-Health applications
In the inpatient rehabilitation program, patients utilise various technological applications such as exercise apps to independently follow their exercise regimen and sensor technology to track their activity levels. As patients transition to home-based rehabilitation, they continue to use these technologies at home for their exercises, engage in remote treatment sessions, or participate in evaluations with the medical team via video calls. Using eHealth aims to promote efficient rehabilitation care and stimulate patient autonomy according to the principle of digital if it can and face-to-face if needed. The appointment was made to provide blended treatment methods, with half of the interaction being face-to-face and the other half occurring digitally through eHealth applications.

5. Mapping the patient's living environment
Before admission to the GR department, the patient's family completes a home inventory form and takes photos or videos of the patient's home. An occupational therapist then visits the patient's home early in the trajectory to assess potential challenges and opportunities at home, determining if the patient needs any assistance or specific training for discharge home and starting HBGR.

6. Stimulating support from informal caregivers

Every month, an educational meeting is organised for informal caregivers, who are provided with a detailed explanation of the entire GR process, including the HBGR trajectory. Also, informal caregivers are invited to attend treatment sessions to receive coaching on guiding patients during training and daily care moments.

7. Collaboration with community care nursing

This collaboration is a crucial aspect of the HBGR trajectory. Community care nurses see the patient most of the time. Two topics were elaborated on during the pilot: i.) the transfer moment home and ii.) education on challenging rehabilitation climate at home.

i. Transfer moment: Smooth and warm transfer home: Just before the transfer home, a warm handover is planned, which involves an online meeting between the patient, the community care nurse, and the GR nurse. At the same time, the multidisciplinary rehabilitation treatment plan, including a transfer letter, will be transferred.

ii. Education on challenging rehabilitation climate at home: The challenging rehabilitation climate is a stimulating environment for the patient that promotes autonomy, encourages greater responsibility for the rehabilitation process, and allows for rehabilitation care while minimising direct assistance (2). Three workshops were organised for community care nurses on supporting and stimulating a patient at home during the rehabilitation process and promoting a challenging rehabilitation climate at home. This is crucial because the challenging rehabilitation climate provided during the inpatient-GR disappears when someone is at home, which is essential to promote progress in the rehabilitation.

8. Rehabilitation coordinator

The role previously known as "case manager" has now been changed to "rehabilitation coordinator" to avoid confusion with this role in dementia care. This rehabilitation coordinator is a professional from the most involved discipline during rehabilitation and will be assigned to patients once their discharge date is known. If community care nursing is involved, they will take on the role of rehabilitation coordinator. The coordinator is the central contact person for the patient and the informal caregivers and will ensure that professional and informal care is being provided as planned.

9. Central planning

The treatments are centrally planned by a separate function outside the MD team to achieve better coordination between the disciplines and save time.

10. Therapy at home unless……

The treatment begins with practical exercises and care at the patient's home, followed by outpatient sessions at the clinic when appropriate. This takes place in consultation with the patient and is tailored to the possibilities.

11. Online multidisciplinary (MD) evaluation meeting

Around three weeks after starting the HBGR trajectory, an online meeting is scheduled for the MD team members, patients, and informal caregivers. This meeting aims to evaluate the progress and rehabilitation needs based on the MD rehabilitation plan.

The following table shows 40 topics gathered and discussed at the first co-creation meeting, divided into the building blocks, structure, process, environment, and outcome.

**Structure**		
1.	Instruction always HBGR unless….	
2.	Education for community care nursing teams, primary care, and informal caregivers	
3.	Transport of a patient from home to the rehabilitation department for outpatient therapy	
4.	Transport of a healthcare professional to the patient at home	
5.	Which disciplines are needed during an HBGR trajectory	
6.	Who are the care chain partners?	
7.	Close cooperation with primary care	
8.	Close cooperation with the social domain	
9.	How to deal with current financial constraints	
10.	EHealth (presence of devices / ICT infrastructure/digitalisation vision/ remote training and image care approach plan)	
11.	Clear overview of who/when/why involved and duration of HBGR (Ysis)	
12.	Visible planning (for all disciplines)	
**Process**		
13.	Central planning	
14.	Interim and final evaluation meeting	
15.	Deployment of homework programmes	
16.	Deployment of technology equipment and apps --> blended unless…..	
17.	Case manager/central contact person	
18.	Collaboration with community care nursing starts during inpatient GR	
19.	Transfer to community care nursing team	
20.	Contact point and contact details community care nursing team	
21.	Fine-tuning the timing of the home visit --> earlier in the trajectory	
22.	timemanagement; planning, uren inzet	
23.	Clarity about the inclusion criteria for HBGR	
24.	Explore more inflow opportunities for HBGR: from home and hospital	
25.	Information provision about HBGR	
26.	Setting up rehabilitation care pathway per diagnosis properly	
27.	Transfer from inpatient to HBGR	
28.	Transfer from HBGR to primary care or finishing HBGR	
**Environment**		
29.	Location treatment tailored to the patient (--> rehabilitation at home unless….)	
30.	Informal caregiver; standard involvement and coaching	
31.	Challenging rehabilitation climate at home	
32.	Providing exercise equipment at home	
**Outcome**		
33.	Shortening length of inpatient stay	
34.	The patient is in control of the HBGR trajectory --> patient autonomy	
35.	More patients are satisfied with the level of recovery	
36.	Less chronic/long-term community care nursing is needed	

37.	The participation level of the patient: the view of patients on their recovery is more positive/better	
38.	Patient's quality of life	
39.	Core set outcome measurements	
40.	Identification of problems and barriers we see during the implementation of HBGR	

Reference

1. Law M, Baptiste S, McColl M, Opzoomer A, Polatajko H, Pollock N. The Canadian occupational performance measure: an outcome measure for occupational therapy. Can J Occup Ther. 1990;57(2):82-7.
2. Tijsen LM, Derksen EW, Achterberg WP, Buijck BI. Challenging rehabilitation environment for older patients. Clin Interv Aging. 2019;14:1451-60.
